# The minipig as a potential model for pedicle screw fixation: morphometry and mechanics

**DOI:** 10.1186/s13018-019-1292-9

**Published:** 2019-08-05

**Authors:** Robert A. Harper, Ferris M. Pfeiffer, Theodore J. Choma

**Affiliations:** 1 Department of Orthopedic Surgery, University of California-Davis, 4860 Y Street, Sacramento, CA 95817 USA; 2 Department of Biological Engineering, University of Missouri, 247 Ag Engineering Building, Columbia, MO 65211 USA; 3 Department of Orthopaedic Surgery, University of Missouri, 1100 Virginia Avenue DC953.00, Columbia, MO 65212 USA

**Keywords:** Bone mineral density, Pedicle screw, Morphometric characterization, Spinal fixation, Biomechanical analysis, Porcine vertebra model

## Abstract

**Background:**

While there are several different animal models for use in the characterization of spinal fixation, none have emerged as a definitive model for comparative studies in spinal fixation methods. The purpose of this study is to establish morphometric data of porcine vertebrae and to characterize the feasibility of pedicle screw fixation in porcine spines for potential comparative human study.

**Methods:**

Four spines from 45 to 50 kg Hanford minipigs were cleaned of soft tissue and analyzed by computed tomography and dual-energy x-ray absorptiometry. Two 5 × 30-mm pedicle screws were placed in each vertebra and tested to failure using a combined moment-load protocol.

**Results:**

Pedicle widths were measured from L6-T5. Widths ranged from 7.15 mm (T6) to 9.24 mm (T14). Posterior cortex to anterior cortex depth ranged from 25.9 to 32.6 mm. Mean bone mineral density was 1.0665 g/cm^2^ (range 1.139–1.016). Force-to-failure demonstrated mean 1171.40 N (+ 115.34).

**Conclusion:**

Our baseline morphometric and compositional data demonstrate that porcine vertebrae can serve as a useful model for comparative studies due to their similar pedicle widths and bone mineral density to the human vertebra. This biomechanical data could provide a baseline comparison for future studies. This study also suggests that the minipig could be a suitable model for comparative studies due to similarities in pedicle width and bone mineral density to the human vertebrae.

## Introduction

There is no generally accepted single animal model for spinal fixation methods. Several different study animals have been used in the characterization of spinal fixation, including sheep [1, 2], cow [3–7], and pig [8], but none has emerged as a gold standard for comparative study. The minipig is an established research animal for comparative human studies in areas as diverse as dermatology [9], endocrinology/diabetes [10], cardiology [11], immunology [12], pharmacology [13], and toxicology [14] due to similarities to human systems. The porcine spine may also be an important model for human spinal fixation and instrumentation techniques. McClain et al. found key similarities of porcine to the human vertebra in their analysis of the morphometry of the L4 vertebra in several large animal species [15]. Dath et al. compiled a database of porcine vertebral measurements—including pedicle width, end plate size, and spinous process size—but limited their measurements to lumbar vertebrae [16] and did not include an important measurement for spinal instrumentation: the screw path length from posterior to anterior cortex, referred to by Krag et al. as “chord length” [17]. Characterization of pedicle isthmus width and chord length for the porcine thoracic spine has not been previously published. As such, comprehensive characterization of porcine vertebral morphometry is necessary for effective human comparison.

If the minipig is to be a useful model for spinal fixation methods, measurements of bone mineral density (BMD) and pedicle screw force to failure in normal porcine spines would be necessary for baseline comparison to future fixation experiments. This leads to the current investigation into the feasibility of using the minipig to assess spinal fixation strategies.

Many previous authors assessing the biomechanics of pedicle screw fixation used pure axial pullout to assess screw interface, but there are many questions about the clinical validity of these simple loading schemes. We have described previously a complex test method that simulates complex in vivo loading for pedicle screw fixation analysis [18].

The goals of this pilot study are twofold: to establish morphometric and compositional data of the minipig thoracic and lumbar vertebrae for potential comparative human study and to characterize the feasibility of pedicle screw fixation in normal minipig spines for comparison with future studies.

## Materials and methods

### Morphometry

Four porcine spines, cleaned of soft tissue, were procured from the Sinclair Research Laboratory Farm (Auxvasse, MO). The spines were from adult male Hanford miniature swine aged 14 to 15 months, weighing 45 to 50 kg, which were sacrificed for reasons unrelated to this particular study. The lumbar regions of the whole vertebral columns were scanned with dual-energy x-ray absorptiometry (DXA) (Hologic, Bedford, MA) to determine BMD. Specimens were then imaged with quantitative computerized tomography (Toshiba American Medical Systems, Tustin, CA) to generate vertebral morphometry. Using Amira (Visage Imaging GmbH, Berlin, Germany) visualization software, transverse pedicle isthmus width and distance from posterior to anterior cortex along the pedicle axis (AC depth) were measured for each vertebra.

### Screw purchase

**Fig. 1:**
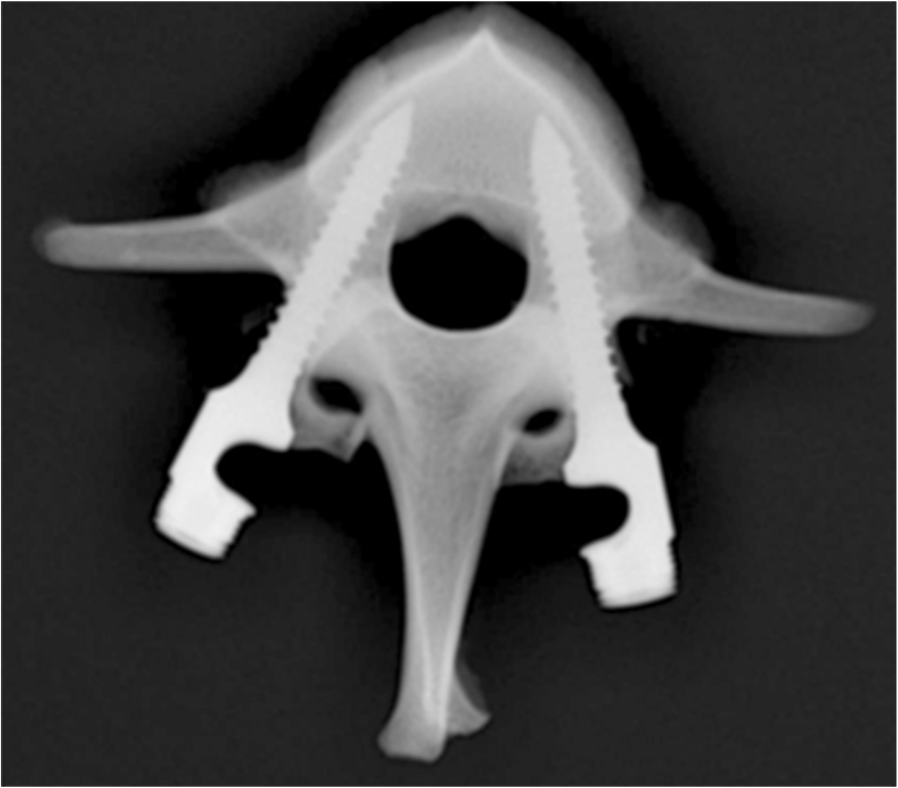
Radiograph of the lumbar vertebra with pedicle screws implanted bilaterally

The vertebrae were separated from connective tissue for instrumentation. A small starting hole (approximately 3 mm) in the posterior cortex was created on each side with a rongeur, exposing cancellous bone. A pilot hole was made with a tapered Lenke probe. A titanium 5 × 30-mm (Synthes USS) fixed pedicle screw (Synthes AG, West Chester, PA) was inserted into each pilot hole (Fig. 1) and a 5.5 × 65-mm titanium rod was inserted into each screw head and secured with an endcap and locking nut, tightened to the manufacturer’s recommended torque.

**Fig. 2:**
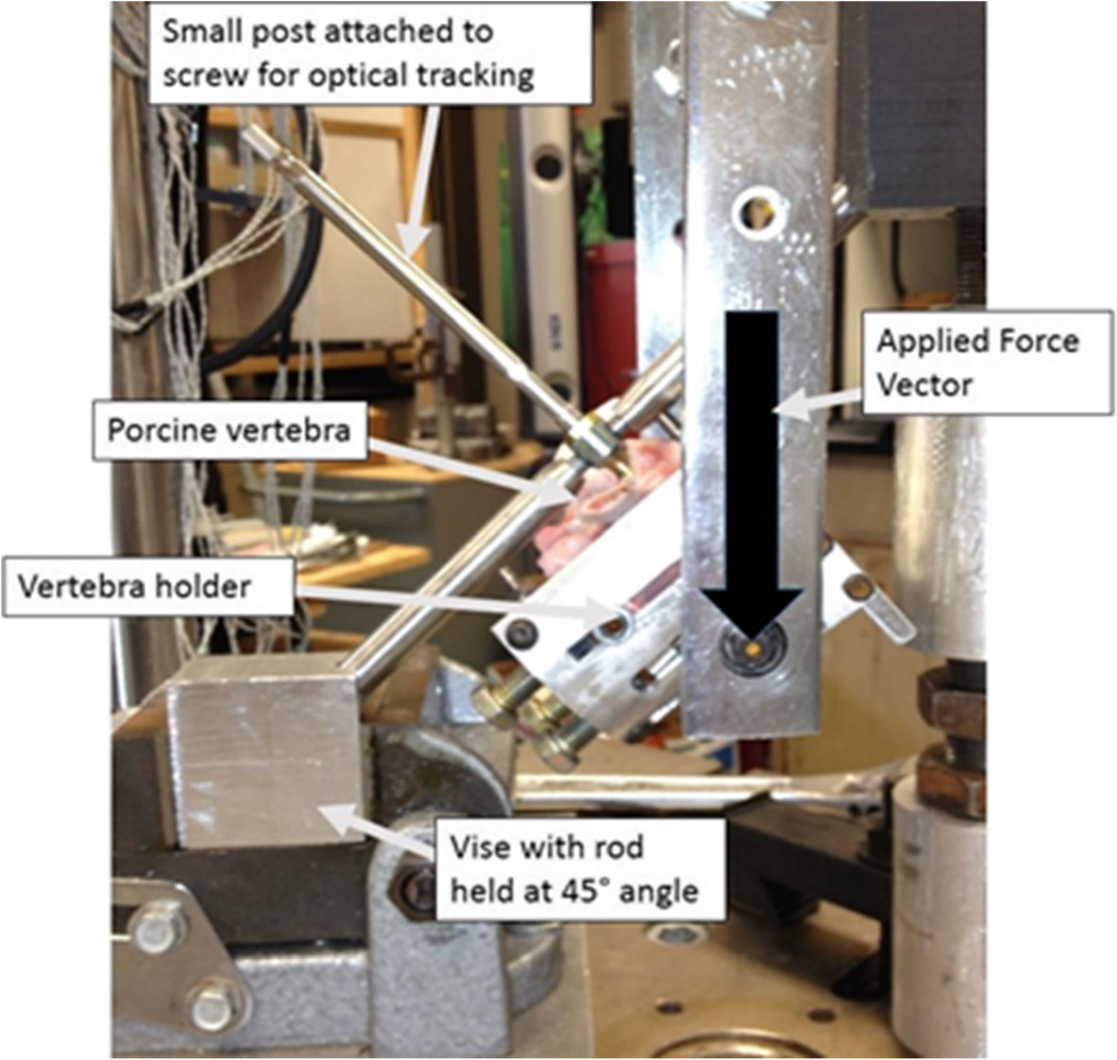
Vertebra with implanted pedicle screw clamped into the apparatus. A titanium rod is inserted into the screw and is secured inferiorly by a vise

The inferior portion of the rod was held securely in an angle vise, *Θ* = 45, and stabilized superiorly to prevent bending. The specimen was held in a custom apparatus which clamped the vertebral body similar to the complex loading previously described (Fig. 2) [1]. The apparatus was attached to a 46.6-cm long pushrod. Loading tests were performed using a servo-hydraulic test machine (Instron 8821S, Norwood, MA). Force was applied through the pushrod at a displacement controlled rate of 5 mm/s until ultimate failure, defined as either fracture of the pedicle or mechanical failure (fracture of plastic deformation) of the screw.

## Results

### Morphometry

**Fig. 3:**
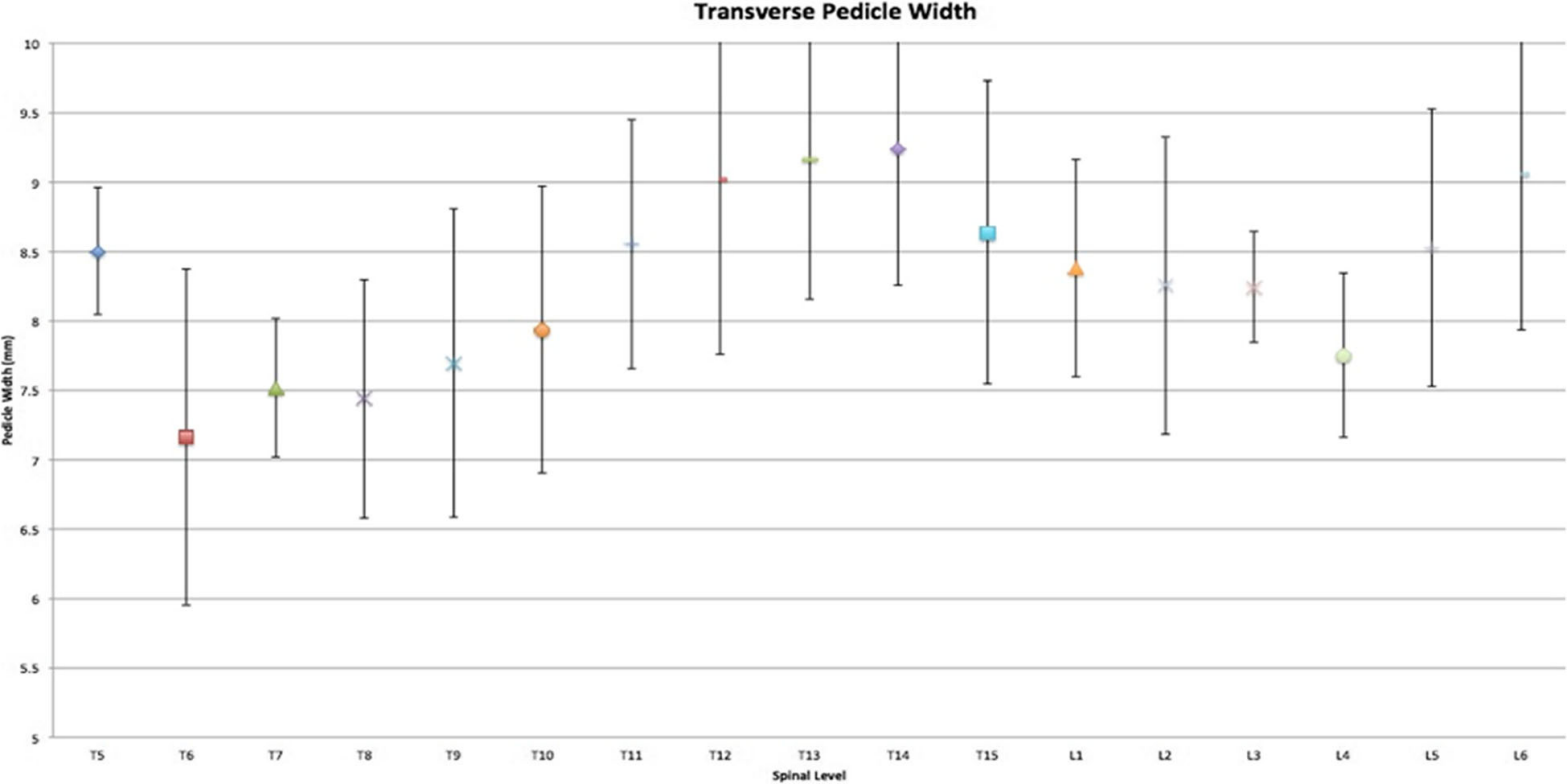
Graph showing the mean transverse pedicle width with standard deviation

**Fig. 4:**
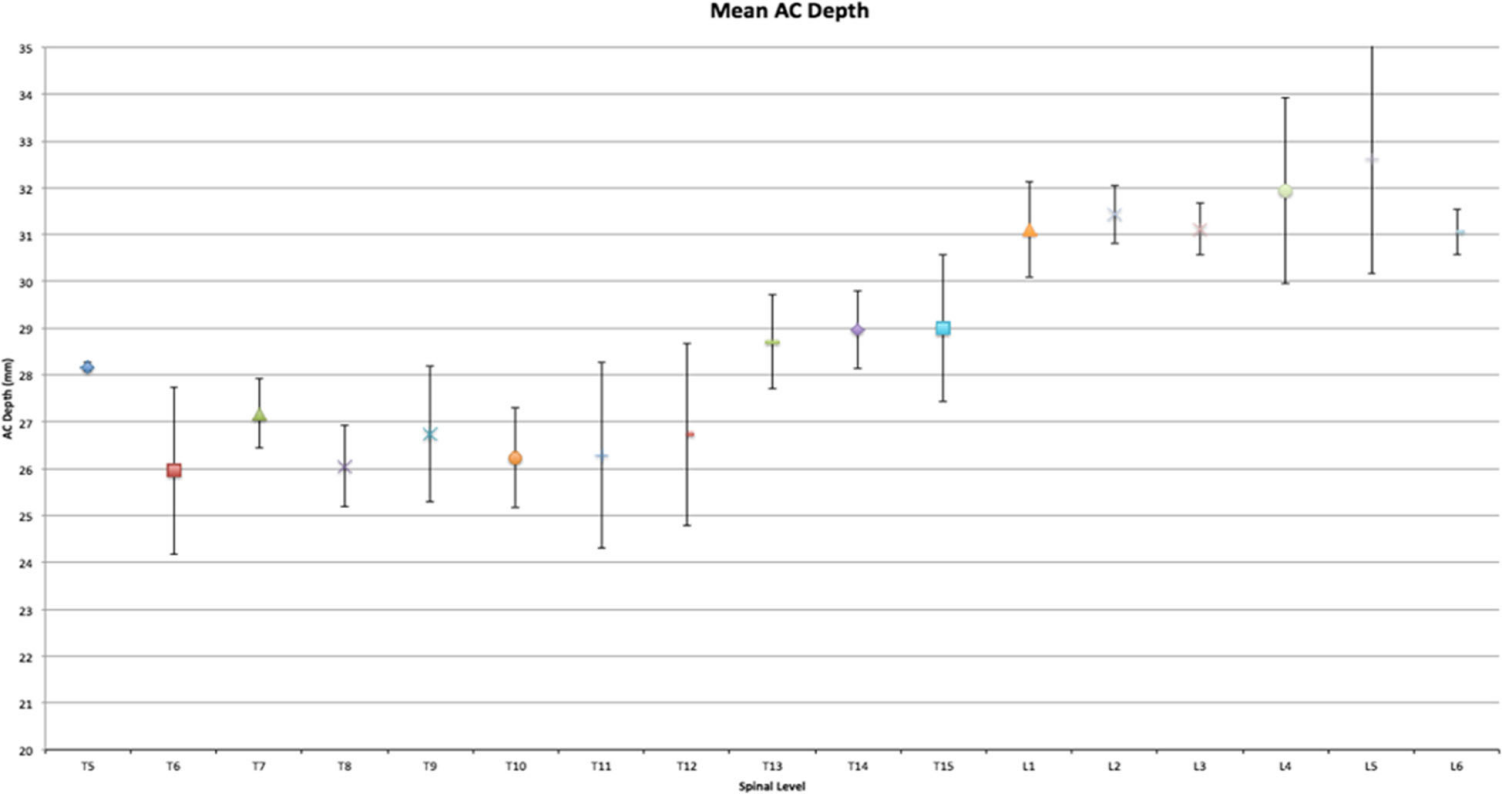
Graph showing the mean AC depth with standard deviation

Mean BMD across all specimens was 1.067 g/cm^2^ (±.0462 g/cm^2^) (Table 1). The widest pedicle isthmus was seen at T14 with a mean value of 9.24 mm (range 7.45 to 10.47 mm). The narrowest pedicle isthmus was seen at T5 with a mean value of 7.16 mm (range 5.56 to 8.59 mm) (Fig. 3). The longest AC depth was measured at L5 with a mean value of 32.61 mm (range 29.44 to 35.5 mm). The shortest was measured at T6 with a mean value of 25.96 mm (range 24.21 to 28.84 mm) (Fig. 4).

**Table 1:** Specimen bone mineral density

Specimen	BMD g/cm^2^
Spine 1	1.139
Spine 2	1.016
Spine 3	1.04
Spine 4	1.071

### Fixation biomechanics

In each case, the failure loads were so high that the screws bent either just previous to or simultaneously with screw failure. Mean force to failure was 1171.40 N (± 115.43) (Table 2). Once we recognized how constant this failure load and mechanism was, we discontinued loading to failure. The consistency of the failure data made it necessary to test only four vertebrae for a total of eight measurements.

**Table 2:** Pedicle screw force to failure specimen bone mineral density

Vertebra	Screw force to failure (*N*)
L2 left	1124.58
L2 right	1208.39
L3 left	1141.36
L3 right	1039.12
L4 left	1405.33
L4 right	1050.24
L5 left	1202.58
L5 right	1199.57

## Discussion

The mean and standard deviation of BMD values for young normal adults have been previously published in the literature. Simmons et al. reported the adult human reference BMD for each of the principal DXA manufacturers. Reported values were as follows: Hologic 1.079 g/cm^2^ (+ 0.110), Lunar 1.200 g/cm^2^ (+ 0.120), Norland Europe 1.085 g/cm^2^ (+ 0.115), and Norland US 1.164 g/cm^2^ (+ 0.162) [19]. The adult Hanford minipig in this study demonstrated a mean BMD of 1.067 g/cm^2^ (± .0462 g/cm^2^), comparable to adult human reference BMD reported in the literature. This similarity is a favorable factor in determining whether the minipig would be a suitable study model. Our findings also agree with others who have investigated porcine BMD.

Lee et al. found similar BMD to our study in their in vitro porcine model of osteoporosis with a mean BMD of 1.189 ± .05 g/cm^2^ [20]. Mitchell et al. reported a mean BMD measurement of 1.057 g/cm^2^ in live, anesthetized, 60 kg pigs [21]. The similarity to the present study helped to mitigate the concern that in vitro DXA measurements of porcine spines cleaned of soft tissue would not accurately represent in vivo BMD values. Although we did not study mineralization, Moskilde et al. found that the porcine skeletal system contains lamellar bone and undergoes trabecular and cortical remodeling in a similar fashion to humans [22]. In a separate study, Moskilde et al. were able to create osteoporosis in the minipig spine through ovariectomy, calcium restriction, and glucocorticoid administration [23]. This raises the prospect that future spinal fixation studies employing Moskilde’s osteoporosis model, compared with the data in our study may elucidate effective strategies for spinal fixation methods in osteoporotic spines. Our BMD analysis allows us to infer that the porcine spine may be a suitable comparison to the human spine.

Along the same lines, some animal model research has focused on coated pedicle screws that may improve fixation at the bone-implant interface [6, 24–26]. Ohe et al. recently implanted 3 types of screws—untreated, sandblasted, and hydroxyapatite (HA)—into 8 Clawn miniature pigs in a 24-week study (6 osteoporosis group, 2 control). They found that the HA coating may reduce the level of loosening of the screws but that absence of increased BMD around the screws was still problematic [26].

**Fig. 5:**
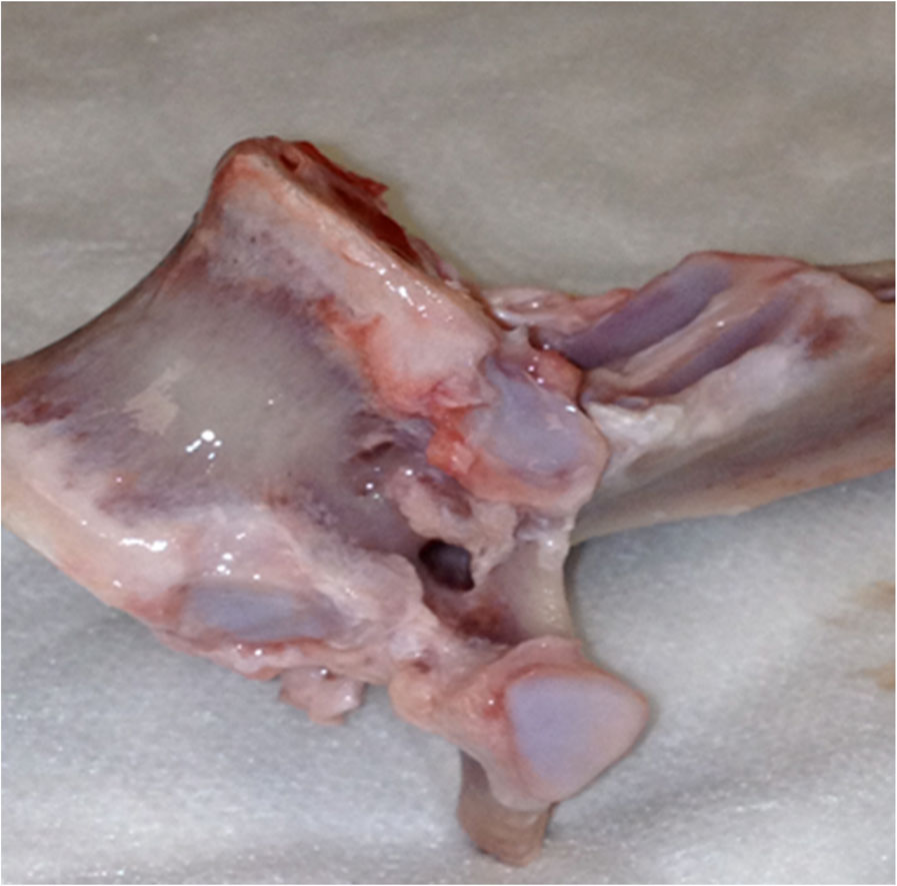
Thoracic vertebra with lateral foramen bisecting the pedicle

The pedicle morphometry data in our study demonstrate that instrumentation with adult human-sized pedicle screws is possible. Lumbar and thoracic vertebrae had similar pedicle isthmus widths to the human widths measured by Zindrick et al. [27]. Mid to upper thoracic pedicles in our specimens demonstrated a perforated pedicle, in which there was a lateral foramen that bisected the pedicle (Fig. 5). This aspect of the spines may preclude pedicle screw instrumentation of that region. Distance from the posterior to the anterior cortex (AC depth) was considerably shorter than that measured in humans by Zindrick et al. [27]. However, most of the lumbar and lower thoracic vertebrae had AC depths greater than 30 mm, which accepted a 5 × 30-mm screw in this study, without ventral perforation. Mid- to upper thoracic vertebrae had AC depths shorter than 30 mm, making pedicle screw instrumentation less feasible without custom-made short screws.

The vertebrae in our study demonstrated an extremely high force to failure. In this pilot study, the titanium rod would often bend before screw failure. By providing enhanced rod reinforcement and defining failure as the relative movement of the screw suggesting displacement within the trabecular meshwork, we were able to achieve consistent force to failure data. Aerssens et al. found that porcine bone had a higher bone mineral content and higher yield stress necessary for trabecular compression than commonly seen in humans [28]. In a human-to-porcine comparison of bone microarchitecture, the porcine spine was shown to have thicker trabeculae [11]. This may require further study to fully characterize and draw comparative conclusions to adult human vertebral bone strength.

Our study has several limitations. The high failure loads exhibited in this study were quite notable, raising the question of whether mechanical findings in the healthy minipig would be directly predictive of implant behavior in humans. These data could be the result of differences in the microarchitecture of the porcine spine or a function of the smaller vertebral bodies. However, even if the force to fixation failure is consistently higher than in the adult human, the relative patterns of fixation between healthy bone and induced osteopenia/osteoporosis may still be very instructive. Another limitation is the shorter AC depth in the mid to upper thoracic vertebrae which precluded pedicle screw instrumentation as our smallest available screw length was 30 mm. Since porcine spines contain up to 15 thoracic and up to 6 lumbar vertebrae, we found 8 to 10 vertebrae from each spine in our study that would accommodate a pedicle screw, more than adequate to conduct studies in spinal fixation. Given the morphometric variation based on spinal levels, it would seem very important that any future studies specify exactly which porcine vertebrae would be used in order to draw conclusions about spinal fixation in humans. A final limitation to this pilot study was the small sample size of four porcine spines. Although our failure data and morphometric analysis in this pilot generated consistent measurements, a larger study with additional specimens may provide additional detail beyond our initial characterization.

## Conclusions

This pilot study suggests that the minipig could be a suitable model for comparative studies due to similarities in pedicle width and BMD to the human vertebrae. These measurements and biomechanical data could provide a useful reference for future studies of spinal fixation methods considering the minipig as a model.

## Data Availability

All the available data analyzed during this study are included in this published article.

## Abbreviations

AC: Anterior cortex
BMD: Bone mineral density
DXA: Dual-energy x-ray absorptiometry
HA: Hydroxyapatite
